# Preserved and Impaired Emotional Memory in Alzheimer’s Disease

**DOI:** 10.3389/fpsyg.2012.00331

**Published:** 2012-09-14

**Authors:** Yanica Klein-Koerkamp, Monica Baciu, Pascal Hot

**Affiliations:** ^1^Laboratoire de Psychologie et Neurocognition, CNRS UMR-5105Grenoble, France; ^2^Université Pierre Mendès FranceGrenoble Cedex, France; ^3^Université de SavoieChambéry Cedex, France

**Keywords:** emotion, memory, Alzheimer’s disease, amygdala

## Abstract

Patients with early atrophy of both limbic structures involved in memory and emotion processing in Alzheimer’s disease (AD) provide a unique clinical population for investigating how emotion is able to modulate retention processes. This review focuses on the emotional enhancement effect (EEE), defined as the improvement of memory for emotional events compared with neutral ones. The assessment of the EEE for different memory systems in AD suggests that the EEE could be preserved under specific retrieval instructions. The first part of this review examines these data in light of compelling evidence that the amygdala can modulate processes of hippocampus-dependent memory. We argue that the EEE could be a useful paradigm to reduce impairment in episodic memory tasks. In the second part, we discuss theoretical consequences of the findings in favor of an EEE, according to which a compensatory mechanism in patients with AD solicits greater amygdala functioning or additional networks, even when amygdala atrophy is present. These considerations emphasize the relevance of investigating patients with AD to understand the relationship between emotion and memory processes.

## Emotional Effects on Preserved and Impaired Memories in Alzheimer’s Disease

Memory impairments are the core of cognitive dysfunctions reported in Alzheimer’s disease (AD; Mori et al., 1997; Petersen et al., 2000). They are sustained by lesions of the medial temporal lobe (MTL), particularly of the hippocampus (Jack et al., 1997; Mori et al., 1997; Simic et al., 1997; Mizuno et al., 2000). Neuroimaging evidence suggests that, in parallel with the development of lesions in the hippocampus, the amygdala undergoes early atrophy in AD (Basso et al., 2006; Horinek et al., 2007; Poulin et al., 2011). The amygdala is strongly implicated in the context of emotional processing (Phelps and LeDoux, 2005) and memory (Cahill et al., 1995, 1996), raising numerous questions about possible impaired mechanisms in AD. While AD studies on emotional processing have sometimes revealed spared emotional abilities (Klein-Koerkamp et al., 2012) and preserved physiological responsiveness to emotion (Smith, 1995; Hamann et al., 2000), an important issue to evaluate could be how emotions modulate memory performance. In the context of normal aging, several studies have reported that emotional content might improve memory performance compared with non-emotional content (emotional enhancement effect: EEE; see; Broster et al., 2012). In this review, we investigate how the EEE on memory performance evolves in healthy older adults (HOA) compared with AD patients.

Findings on the effect of emotion on memory in AD have led to discrepant results, with some studies reporting an EEE or a beneficial effect of emotion on memory (i.e., the emotional material is more accurately recalled than neutral material), and others reporting no emotional advantage (i.e., emotional material is less recalled than neutral material, or equivalently recalled). Thus, in this report, we review these effects, along with factors that could modulate the EEE: the participant’s characteristics and the emotional task design (i.e., emotions, stimuli, and procedure used). We further compare in detail these emotional effects in AD patients and HOA with respect to the differences in their overall memory performance. The literature that provides the basis for this review was obtained by searching PubMed, PsycARTICLES, PsychINFO, and Psychology and Behavioral Sciences Collection databases for English language articles containing the key terms “Emotion*” AND “Memory” AND “Alzheimer” in the title and/or the abstract and/or the keywords. No restrictions were placed on the year, with all articles up to May 2012 included. Relevant papers from the reference lists of identified papers were also reviewed. Given the focus on AD patients, only studies with samples of people with this dementia were included. In addition, only those studies were considered that included the diagnostic criteria used to identify AD patients [criteria recommended either by the National Institute of Neurological and Communicative Disorders and Stroke and the AD and Related Disorders Association (NINCDS/ADRDA; McKhann et al., 1984)], or by the fourth edition of the *Diagnostic and Statistical Manual of Mental Disorders* (DSM-IV, 1994). Two studies were excluded because the patient group did not differentiate between individuals with AD and those with mixed dementia (Blessing et al., 2006, 2010). Finally, the study had to include at least one explicit measure of the emotional effect on memory performance (i.e., a comparison of memory performance between an emotional and a neutral event). As a result of this last criterion, the studies included in this review dealt mostly with declarative memory. Ultimately, 22 studies concerning the EEE on memory performance in AD were eligible for inclusion in the present review (Table 1).

**Table 1:** **Comparative review of studies investigating the EEE on memory**.

Study	Participants	Emotions and Stimuli	Type of Encoding	Tasks	Emotional Assessment	Presence of EEE	Group Difference on Memory Performance
Abrisqueta-Gomez et al. (2002)	AD = 16; mean age = 70; m/f = 7/9; MMSE = 19,6; HOA = 19; mean age = 67; m/f = 7/12; MMSE = 28,9	Pleasant/unpleasant and neutral scenes	Intentional	Recognition (after 30 min)		Yes HOA (pleasant – unpleasant); no AD	AD < HOA
				Emotional categorization	AD = HOA		
Boller et al. (2002)	AD = 10; mean age = 75; m/f = 5/5; MMSE = 19,6; HOA = 12; mean age = 75; m/f = 8/4; MMSE = 28,8	Happy, sad, and neutral stories	Intentional	Immediate and delayed free recall (after 10 min)		Yes HOA (happy – immediate recall); Yes AD (happy and sad – immediate recall); No HOA (delayed recall); No AD (floor effects – delayed recall)	AD < HOA
				Questionnaire		No HOA (ceiling effects); yes AD (sad and happy)	AD < HOA
				Emotional categorization	AD < HOA		
Borg et al. (2011)	AD = 14; mean age = 80; m/f = 4/10; MMSE < 24; HOA = 14; mean age = 78; m/f = 5/9; MMSE > 27	Negative and neutral scenes (IAPS)	Intentional	Recognition (visual memory task)		Yes HOA; yes AD	AD = HOA
				Recognition (location task – memory binding)		No HOA; no AD	AD < HOA
Brueckner and Moritz (2009)	AD = 36; mean age = 72; m/f = 16/20; MMSE = 24; HOA = 20; mean age = 69; m/f = 8/12	Thematic word lists (depression; delusion; positive; neutral)	Intentional	Recognition		Yes HOA (true recognition); No AD (true recognition); Yes HOA (false recognition); Yes AD (false recognition)	AD < HOA (true recognition)
Budson et al. (2006)	AD = 19; mean age = 76; m/f = 9/10; MMSE = 23; HOA = 19; mean age = 73; m/f = 7/12; MMSE > 27	Thematic word lists (emotional and non-emotional)	Intentional	Recognition (after 5 min)		Yes HOA (true recognition); No AD (true recognition); No HOA (false recognition); No AD (false recognition)	AD = HOA (true recognition); AD > HOA (false recognition)
Fleming et al. (2003)	AD = 25; mean age = 75; MMSE = 21 HOA = 19; mean age = 70	Thematic word lists (negative, positive, neutral)	Intentional	Free recall		No HOA; yes AD (negative)	AD < HOA
Gallo et al. (2010)	AD = 18; mean age = 77; m/f = 7/11; MMSE = 23,9; HOA = 18; mean age = 72; m/f = 6/12; MMSE = 28,8 (HOA and AD results reported on the same experimental condition)	Negative, positive, neutral words and scenes (IAPS)	Intentional	Recognition		No HOA (true recognition); No AD (true recognition); Yes HOA (positive – false recognition); yes AD (positive – false recognition)	AD < HOA
				Arousal rating	AD = HOA (emotional > neutral)		
Hamann et al. (2000)	AD = 12; mean age = 71; m/f = 5/7; MMSE = 21,5; HOA = 12; mean age = 70; m/f = 3/9; MMSE = 29,2	Negative, positive, neutral scenes (IAPS)	Incidental	Free recall (immediately or after 2 weeks for HOA; immediately after for AD)		Yes HOA (positive and negative); yes HOA (2 weeks’ delay – positive + negative); yes AD (positive)	AD < HOA; AD = HOA (2 weeks’ delay)
				Recognition		Yes HOA (negative); no AD	AD = HOA
				Arousal rating	AD = HOA (emotional > neutral)		
Kalenzaga et al. (2012)	AD = 22; mean age = 83; m/f = 2/20; MMSE = 18,1; HOA = 18; mean age = 85; m/f = 2/16; MMSE = 27,5	Negative, positive, neutral words	Intentional	Recognition (after 10 min for HOA; immediately after for AD)		No HOA; yes AD (positive)	AD < HOA
				Remember – know paradigm		Not determined (remember); no HOA; no AD (know responses)	AD < HOA
Kazui et al. (2000)	AD = 34; mean age = 71; m/f = 7/27; MMSE = 22,5; HOA = 10; mean age = 70; m/f = 3/7; MMSE = 28,6	Arousing (negative) and non-arousing stories with pictures	Intentional	Questionnaire (after 2 weeks) Emotional rating	AD = HOA emotional > neutral	Yes HOA; yes AD	AD < HOA
Kazui et al. (2003)	AD = 56; mean age = 72; m/f = 14/42; MMSE = 23,3; no HOA	Arousing (negative) and non-arousing stories with pictures	Intentional	Questionnaire (after 2 weeks)		Yes AD	
Kensinger et al. (2004)	AD = 80; mean age = 71; m/f = 33/47; MMSE = 23,2; HOA (10 min delay) = 33; mean age = 71; m/f = 17/16; MMSE = 29,4; HOA (24 h delay) = 18; mean age = 68; m/f = 8/10; MMSE = 29,1	Negative and neutral stories	Intentional	Immediate and delayed free recall (after 10 min for AD and HOA [10 min delay] or 24 h for HOA [24 h delay])		Yes HOA (10 min delay – immediate and delayed recall); yes HOA (24 h delay – immediate and delayed recall); no AD (immediate and delayed recall)	AD < HOA (10 min delay – immediate and delayed recall); AD < HOA (24 h delay – immediate and delayed recall); AD = HOA (24 h delay – delayed recall)
				Immediate and delayed recognition (after 10 min for AD and HOA [10 min delay] or 24 h for HOA [24h delay])		Yes HOA (10 min delay – delayed recall); no HOA (10 min delay – immediate recall); yes HOA (24 h delay – delayed recall); no HOA (24 h delay – immediate recall); no AD (immediate and delayed recall)	AD < HOA (10 min delay – immediate and delayed recall); AD < HOA (24 h delay – immediate and delayed recall); AD = HOA (24 h delay – delayed recall)
				Valence and arousal rating	AD = HOA (negative > neutral)		
Kensinger et al. (2002)	AD = 13; mean age = 75; HOA = 20; mean age = 73	Positive, negative, neutral pictures	Intentional	Recall		Yes HOA (positive and negative); no AD	AD < HOA
		Positive, negative, neutral words		Recall		Yes HOA (positive and negative); no AD	AD < HOA
		Neutral words in a positive, negative, or neutral context (sentence)		Recall		No HOA; no AD	AD < HOA
		Neutral or negative words		Recognition (after 5 min)		Yes HOA (negative); no AD	AD < HOA
		Neutral words in a negative or neutral context (sentence)		Recognition (after 5 min)		No HOA; no AD	AD < HOA
Moayeri et al. (2000)	AD = 28; mean age = 76; MMSE = 19,6; HOA = 16; mean age = 71; MMSE = 29	Arousing (negative) and non-arousing stories with pictures	Intentional	Recognition and questions (after 5 min)		No HOA (ceiling effect); yes AD (negative)	AD < HOA
Nashiro and Mather (2011)	AD = 18; mean age = 72; m/f = 11/7; HOA = 18; mean	Arousing (positive and negative) and	Incidental	Free recall		Yes HOA (arousing – positive); yes AD (arousing – positive)	AD < HOA
	age = 72; m/f = 6/12	non-arousing scenes (IAPS)		Recognition (location task – memory binding)		Yes HOA (arousing – positive and negative); yes AD (arousing – positive and negative)	AD < HOA
				Recognition		Yes HOA (arousing – positive and negative); yes AD (arousing – negative)	AD < HOA
				Recognition (location task – memory binding)		Yes HOA (arousing – positive and negative); yes AD (arousing – positive and negative)	AD < HOA
Nieuwenhuis-Mark et al. (2009)	AD = 20; mean age = 83; m/f = 17/3; MMSE = 16,2; HOA = 38; mean age = 81; MMSE = 27,4	Positive, negative, neutral words	Intentional	Free recall		Yes HOA (positive and negative); yes AD (positive and negative)	AD < HOA
Perrin et al. (2012)	AD = 15; mean age = 80; m/f = 9/6; MMSE = 24,6; HOA = 15; mean age = 76; m/f = 7/8; MMSE = 28,1	Positive, negative, neutral pictures (with negative, positive, and neutral sound context: dialogs)	Intentional	Free recall (after 3 min)		Yes HOA (positive sound context); no AD (sound context); yes HOA (positive pictures); yes AD (positive pictures)	AD < HOA
				Questionnaire (gist and detail)		Yes HOA (positive and negative pictures for gist); yes AD (positive and negative pictures for gist); no HOA (sound context – for gist and detail); no AD (sound context – for gist and detail)	
				Emotional rating	AD = HOA (positive > neutral > negative)	
Satler et al. (2007)	AD = 10; m/f = 5/5; HOA = 10; m/f = 3/7	Arousal (negative) and neutral stories	Intentional	Questionnaire (after 2 weeks)		No HOA; yes AD (negative)	AD < HOA
				Emotional rating	AD ≠ HOA		
Satler et al. (2009)	AD = 14; mean age = 75; m/f = 6/8; HOA = 10; mean age = 70; m/f = 6/4	Arousal (negative) and neutral video clips	Intentional	Questionnaire (after 2 weeks)		No HOA (ceiling effect); no AD	AD < HOA
				Emotional rating	AD ≠ HOA		
Schultz et al. (2009)	AD = 20; mean age = 70; m/f = 10/10; MMSE > 20; HOA = 20; mean age = 66; m/f = 10/10 (results reporting on global HOA group – not on years of schooling subdivisions)	Negative, positive, neutral scenes (IAPS)	Incidental	Immediate and delayed free recall (after neuropsychological battery assessment)		Yes HOA (pleasant and unpleasant – immediate recall; delayed recall); yes AD (pleasant and unpleasant – immediate recall); yes AD (pleasant – delayed recall)	AD < HOA
				Recognition		Not determined	AD < HOA
				Pleasantness and valence rating	AD ≠ HOA		
Sundstrom (2011)	AD = 20; mean age = 73; m/f = 10/10; MMSE = 19,9; HOA = 20; mean age = 71; MMSE = 27,4	Emotional objects (gifts) and non-emotional objects (gifts)	Incidental	Free recall		No HOA; yes AD	AD < HOA (for both emotional and non-emotional)
				Recognition		No HOA (ceiling effect); no AD	AD < HOA (for both emotional and non-emotional)
Werheid et al. (2011)	AD = 18; mean age = 76; m/f = 5/13; MMSE = 24,6; HOA = 18; mean age = 75; m/f = 9/9; MMSE = 29,5	Happy, angry, neutral faces	Intentional	Recognition		Yes HOA (anger); yes AD (anger)	AD = HOA (accuracy)
				Emotional categorization	AD = HOA (anger > happy > neutral)		

*If not indicated, recall and recognition were performed immediately. AD: Alzheimer’s disease patients; HOA: healthy older adults; IAPS: International Affective Picture System (Lang et al., 1999); AD < HOA means that performances of AD patients were lower than those of HOA. AD = HOA means that performance was equivalent across groups*.

The magnitude of the EEE on hippocampus-dependent memory (declarative memory) has been assessed in AD patients mostly by using recall and recognition tasks with intentional encoding. Additionally, various sets of emotional materials have been used: emotional short stories with illustrated pictures, visual scenes, video clips, word lists, and objects (Table 1). Although memory was typically found to be heavily impaired in patients with AD, the beneficial effect of emotions (EEE) was repeatedly demonstrated in patients by using various types of tasks and materials (Kazui et al., 2000, 2003; Boller et al., 2002; Fleming et al., 2003; Nieuwenhuis-Mark et al., 2009; Schultz et al., 2009; Borg et al., 2011; Nashiro and Mather, 2011; Werheid et al., 2011; Perrin et al., 2012). This EEE was sometimes retrieved for discrete emotional categories, such as only positive emotions (Hamann et al., 2000; Kalenzaga et al., 2012; Perrin et al., 2012), but also for both positive and negative emotions (Moayeri et al., 2000; Satler et al., 2007; Werheid et al., 2011). However, in contrast to these beneficial effects, a reduced EEE has also been reported, even when patients viewed the same emotional stimuli or performed very similar tasks to those used in studies reporting a preserved EEE (Hamann et al., 2000; Abrisqueta-Gomez et al., 2002; Kensinger et al., 2002, 2004; Budson et al., 2006; Brueckner and Moritz, 2009; Perrin et al., 2012). Some researchers (Kensinger et al., 2004; Budson et al., 2006; Kensinger, 2006) have suggested that the EEE on memory could be disrupted in AD. We argue that several factors could intervene in the presence or absence of EEE in AD. Intrinsic differences in memory functioning between controls and AD patients could lead to difficulties in raising an EEE. Researchers have also proposed that the retrieval instructions (e.g., recollection vs. recognition tasks; Kensinger et al., 2002, 2004; Sundstrom, 2011) or the type of emotional stimuli (Kensinger, 2006; Nashiro and Mather, 2011; Sundstrom, 2011) might modulate the magnitude of EEE in AD patients.

Several studies found that the EEE was present in AD patients despite it not being present in the control group (Moayeri et al., 2000; Boller et al., 2002; Fleming et al., 2003; Sundstrom, 2011; Kalenzaga et al., 2012), or, inversely, that it was present in HOA but not in the AD population (Boller et al., 2002; Kensinger, 2006). Some authors stressed that when the task to be performed is too easy for HOA, or too difficult for patients, the EEE is more likely to be obscured by a ceiling effect or a floor effect, respectively. These effects could result from overall between-group (AD vs. HOA) differences in memory functioning, which could overshadow the genuine emotional effect. For example, HOA have performed perfectly in recognition tasks (Moayeri et al., 2000; Sundstrom, 2011) or questionnaires (Boller et al., 2002), as these tasks are supposed to be easier for controls than patients. Similarly, previous studies have established that recognition memory was typically very high in HOA when they were tested immediately (Fleming et al., 2003) and that AD patients were particularly impaired in tasks involving a delay, leading to floor effects (Boller et al., 2002). Some studies offered modifications to address these ceiling and floor issues. For example, the study by Werheid et al. (2011) used a paradigm in which the presentation of the stimuli was repeated three times only in the AD group. This study showed that three repetitions permitted patients to benefit from emotional information to improve their memory performance. Further, Kalenzaga et al. (2012) offered a reduced time delay for AD patients compared with HOA. In addition, Hamann et al. (2000) and Kensinger et al. (2004) used two different control populations, one with the same memory task delay as for AD patients and the second with 2 weeks or 24 h of additional delay. The study in which the delay was 24 h longer did not allow equalization of overall memory performance and showed no EEE in the AD group (Kensinger et al., 2004), whereas the other study with 2 weeks of additional delay showed between-group memory performance and an EEE that was similar in HOA and AD patients for positive emotions (Hamann et al., 2000). The confusing pattern of EEE on declarative memories in AD could thus result from the complex interaction between changes in memory and emotion processes. The absence of an EEE in AD was thought by some to reflect the fact that emotion could not interfere with memory, since the disease severely impairs the memory system (Borg et al., 2011). This issue reflected the potential influence of confounding variables (Klein-Koerkamp et al., 2012). Rather than being a deficit in the impact of emotion on memory processing, however, the absence of an EEE could represent a deficit in overall cognitive performance (e.g., short-term memory, verbal abilities, semantic memory, executive functions, visuo-spatial abilities). Several authors argued that the presence of an EEE could result in access to cognitive abilities, such as executive functions (Borg et al., 2011; Broster et al., 2012). Knowing the influences of emotions on executive control (for review see Cohen and Henik, 2012, in this Research Topic), emotional memory enhancement would then be affected in individuals with impairments in executive functions. These considerations point out the need to consider cognitive deficits when exploring the EEE on memory in AD.

Further, we argue that distinguishing the differences in retrieval instructions (recollection versus recognition tasks) could be critical in explaining the EEE discrepancies in AD. Indeed, there is evidence that recollection tasks are more likely to induce an EEE in healthy aging (Ochsner, 2000; Talarico et al., 2004). Although recollection and recognition tasks both involve remembering specific details (e.g., contextual information) of an encoded episode, recollection tasks require a greater engagement of episodic memory (de Vanssay-Maigne et al., 2011). During recognition, processes of familiarity detection may compensate for episodic memory difficulties (Atkinson and Juola, 1974; Mandler, 1980). Familiarity refers to the ability to remember that an episode has been encountered previously when no other contextual information about it is available (Gardiner et al., 1998). In the context of emotional memory, the separation of studies by the function of their retrieval instructions suggests that the EEE remains preserved in patients with AD when recollection tasks are performed (Boller et al., 2002; Fleming et al., 2003; Nieuwenhuis-Mark et al., 2009; Nashiro and Mather, 2011; Perrin et al., 2012) rather than when recognition tasks are performed (Abrisqueta-Gomez et al., 2002; Kensinger et al., 2002, 2004; Budson et al., 2006; Brueckner and Moritz, 2009; Gallo et al., 2010). This effect of recognition vs. recollection was also shown when AD patients could not create an elaborate conscious encoding strategy during incidental encoding (Hamann et al., 2000; Schultz et al., 2009; Sundstrom, 2011). Studies have consistently shown that, whereas recollection is sustained by the hippocampus area, recognition is associated with activities in the perirhinal cortex (for reviews, see Brown and Aggleton, 2001; Diana et al., 2007; Eichenbaum et al., 2007; Skinner and Fernandes, 2007). This distinction is crucial because current theoretical models of the EEE have proposed that emotion influences on declarative memory are sustained by functional connections between the amygdala and hippocampus (Cahill et al., 1995; McGaugh et al., 1996; Cahill and McGaugh, 1998; McGaugh, 2004; Phelps, 2004; Labar and Cabeza, 2006). Dolcos and collaborators (2005) demonstrated in particular that participants elicited greater activity in the amygdala, hippocampus, and entorhinal cortex when they successfully retrieved emotional stimuli than when they retrieved neutral pictures. Most importantly, in the amygdala and hippocampus, the activity for emotional pictures was greater for recollection than for recognition (familiarity; Dolcos et al., 2005), suggesting that successful retrieval of emotional items was related to an amygdalo-hippocampal interaction for recollection tasks. Although influences of amygdale activity during the encoding of emotional faces have been demonstrated in other brain areas (Kilpatrick and Cahill, 2003; Sergerie et al., 2005), findings have mainly been obtained for hippocampus-dependent declarative memory. In patients with AD, the successful recollection of emotional cues supports the concept that the interaction within MTL structures is partly preserved, even when the hippocampus and amygdala volumes are partially reduced. Results from the study by Mori et al. (1999) confirmed a significant correlation between the amygdalar volume of patients with AD and their personal memory but not between amygdalar volume and their factual knowledge about the Kobe earthquake. This suggests that in impaired memory systems, emotional charge could allow individuals to reduce deficits specifically for strictly episodic memory.

Another point considered to be critical in the elicitation of an EEE in AD is the type of emotional stimuli. It has been proposed that differences in stimulus properties, such as exposure time, tactile richness, and self-reference (whether the stimuli relates to oneself or not), might be the reason for the contradictory results of the EEE in AD (Sundstrom, 2011; Kalenzaga et al., 2012). By using objects that were emotionally connoted as gifts, Sundstrom (2011) demonstrated that the emotional load was increased, leading to the generation of an EEE in AD patients. The author then showed, by using a tactile self-reference dimension (the participant received a gift), that the gifts were better recalled than the non-gifts. In addition, in a study by Kalenzaga et al. (2012), the subjects had to perform a recognition task of emotional vs. neutral adjective traits and then had to characterize during encoding the extent to which the adjective described themselves (self-reference encoding). Results showed that this stimuli encoding strategy led to an EEE, in particular for negative adjectives. Thus, it can be expected that AD patients’ attention is attracted by the emotional valence of material that is potentially congruent or emotionally related to themselves (Kalenzaga et al., 2012). This consideration could be applied in the context of flashbulb memories, which are characterized by an enhanced memory for highly emotionally charged situations that have been experienced (Ikeda et al., 1998; Budson et al., 2004). Japanese patients with AD were more likely to be able to recall their emotional experience during the Kobe earthquake than they were to recall the magnetic resonance imaging scan that they underwent at about the same time. Further, Budson et al. (2004) found that patients with AD retained more personal than factual information about their experience during the events of September 11, 2001. Taken as a whole, these findings suggest that the use of different emotional stimuli could lead to different emotional loads, which might potentially generate an EEE, provided that the intensity and the self-reference of the stimulus are high enough.

In sum, the presence or the absence of an emotional effect on declarative memory could depend on several factors: the presence of severe cognitive decline (e.g., memory), the retrieval instruction, and the emotional stimulus (e.g., emotional load, self-reference). A large number of studies have found an EEE on memory in AD even when cautiously controlling the between-group difference in memory performance (Hamann et al., 2000; Werheid et al., 2011; Kalenzaga et al., 2012), thus reinforcing the beneficial effect of emotion on an AD patient’s memory losses. Emotions could convey a conceptual representation that seems to remain partly accessible in these patients. Factors related to the task could also be used to reinforce the emotional trace in memory. The elicitation of an enhancement effect on declarative memory, which declines dramatically in AD, raises the potential benefits of using emotional stimuli in rehabilitations programs. Emotional cues could be a promising way to elaborate therapeutic interventions.

Outside the considerations of EEE on declarative memory, emotional influences in AD could also increase in the context of non-declarative memory (i.e., implicit memory). These emotional influences have not, however, been associated with an EEE, as no strict assessment of memory performances has been done that compared an emotional with a neutral stimulus. Several AD studies have investigated memory implicitly in the context of affective learning (Blessing et al., 2006, 2010), emotional priming (Quoniam et al., 2003; Labar et al., 2005; Garcia-Rodriguez et al., 2009), and fear conditioning (Hamann et al., 2002; Hoefer et al., 2008). The study of Blessing and coworkers (2006) showed that patients’ affective ratings of neutral faces were systematically altered by the biographical information (pleasant or aversive stories) that was previously associated with the face. These authors suggested that implicit affective dispositions were relatively intact in dementia. Similarly, preservation of emotional priming in AD has been suggested, since emotional categorization was more accurate for previous emotional priming than for neutral priming (Labar et al., 2005; Garcia-Rodriguez et al., 2009). On the other hand, AD patients have presented deficits in the acquisition of fear conditioning responses, although normal reactivity to the aversive stimulus was found (Hamann et al., 2002; Hoefer et al., 2008).

Researchers have stressed that the pattern of spared and impaired types of non-declarative and declarative memory follows the pattern of brain regions affected by AD (Hamann et al., 2002; Blessing et al., 2006; Garcia-Rodriguez et al., 2009). Declarative memory is sustained by MTL structures, including the hippocampus and entorhinal cortex, which are first affected in AD. The amygdala was suggested to be a critical structure for the establishment of conditioned responses (Ledoux, 1992; Debiec et al., 2010) and emotional memory (Cahill et al., 1995). This brain structure undergoes pathological changes relatively early in AD (Poulin et al., 2011). In contrast, the visual and sensory cortical areas engaged by visual priming are usually spared in the early stage of the disease (Schacter and Badgaiyan, 2001; Blessing et al., 2006), suggesting that implicit emotional memory could be partly preserved in AD (Garcia-Rodriguez et al., 2009). These considerations point, however, to the notion that pathological lesions (e.g., in the amygdala) result in functional impairments, which is not always admitted by the scientific community (Dickerson et al., 2004; Wright et al., 2007). It is thus necessary to unravel how changes in limbic structures could modulate an emotional impact in AD.

### AD neuropathological changes within the amygdala and their relation to the impact of emotions on memory

Alongside reports about the reduction in hippocampus volume, reports about the atrophy of other limbic areas have been published from the start of investigative work on the AD brain (Herzog and Kemper, 1980; Tsuchiya and Kosaka, 1990; Scott et al., 1991, 1992; Arriagada et al., 1992). In particular, several neuroimaging studies have focused on amygdala lesions (Cuenod et al., 1993; Lehericy et al., 1994; Mori et al., 1997, 1999; Krasuski et al., 1998; Basso et al., 2006; Horinek et al., 2007; Wright et al., 2007; Schultz et al., 2009; Cavedo et al., 2011; Poulin et al., 2011), suggesting that reduction in its volume could be similar to hippocampus atrophy (Killiany et al., 1993; Mizuno et al., 2000; Barnes et al., 2006; Schultz et al., 2009). A reduction in amygdala volume may have dramatic consequences for emotion processing in AD, since this structure is strongly involved in numerous emotional processes (Phelps and LeDoux, 2005; Phelps, 2006; Pessoa and Adolphs, 2010). In a recent meta-analysis of the AD population, we reported that the recognition of amygdala-dependent emotions is consistently impaired across studies (Klein-Koerkamp et al., 2012). In relation to memory, considerable supporting evidence in animal (Maren, 2001) and human studies (Hamann, 2001; McGaugh, 2004; Phelps and LeDoux, 2005; Phelps, 2006) suggests that the amygdala is able to modulate encoding, consolidation, and retrieval of emotional materials by increasing hippocampus activity. Insights from another model indicate that the amygdala could be a specific structure for emotional memorization (Ledoux, 2000). Consequences of specific amygdalar atrophy have been assessed in several neuropsychology studies of patients with bilateral and symmetrical calcification of the amygdalar complex [Urbach-Wiethe (UW) disease]. These works have confirmed that emotional memory disruption could result from amygdalar damage because patients with UW had a reduced EEE (Adolphs et al., 1997), whereas patients with amnesia whose amygdala was spared (but who had damage to other MTL regions) had intact emotional memory (Hamann et al., 1997). It has since been proposed that lesions in the amygdala observed in the early phase of AD could be sufficient to disturb this emotional process (Kensinger, 2006).

The assessment of the EEE on declarative memory in patients with AD and UW, offering a model of amygdala neuropathological lesions, has, however, provided divergent findings. Whereas a deficit in EEE acquisition is observed in UW patients (Adolphs et al., 1997), it is not systematically the case in AD patients. Related to the amygdala, the major difference between these two populations is that patients with UW have complete calcification of both amygdales (for a detailed neuroanatomy description; Tranel and Hyman, 1990), whereas the level of amygdala atrophy in AD varies from 14 to 60% (Scott et al., 1991; Cuenod et al., 1993; Jack, 1997; Cavedo et al., 2011). In addition, a recent study suggests that, rather than affecting the whole structure, amygdalar atrophy in patients with AD affects mainly the lateral and basolateral ventromedial regions (Cavedo et al., 2011). Thus, according to the notion that pathological lesions result in functional impairments, these considerations emphasize that emotional memory deficits will be moderate in AD. Nevertheless, the hypothesis that localized amygdala lesions in AD are responsible for changes in EEE acquisition remains an open question, as (1) the functional role of the amygdala subregions in emotional memory is largely unknown, and (2) both preservation and disruption effects are reported in AD studies. This is inconsistent with the notion that proportional effects exist between pathological lesions and the functionality of the amygdala, which raises critical issues related to amygdala functioning in AD.

To date, very few works have brought new information to light regarding these issues, with most imaging studies investigating memory effect without emotionally arousing information (Grady et al., 2001; Rosenbaum et al., 2010). In these studies, functional connectivity data were obtained in patients with AD engaged in a delayed match-to-sample face recognition task (working memory task) of familiar and unfamiliar items. Results showed increased connectivity between the left amygdala and the neighboring and inferior prefrontal regions in AD compared with that in HOA (Grady et al., 2001; Rosenbaum et al., 2010). This pattern of altered connectivity was not clearly determined. As amygdala activation has consistently been reported during affective tasks, the authors suggested that the emotional content of the faces was incidentally processed to a greater degree by the patients than by the controls (Grady et al., 2001). Some authors have also proposed that the pattern of prefrontal involvement in AD represents an inhibitory system that suppresses emotional responses elicited by the faces, which is irrelevant to the task (Rosenbaum et al., 2010). This pattern of enhanced prefrontal activity (in particular the dorsomedial part of the prefrontal cortex) involved in regulatory mechanisms has been also reported in the context of healthy aging. It was suggested that older adults showed a greater prefrontal activity compared to young adults due to additional cognitive control involved in decoding and/or regulating negative emotions (Ebner et al., 2012). In the context of AD, researchers suggested, nevertheless, that this prefrontal activity reflected a compensatory mechanism, involving the amygdala and prefrontal networks to a greater extent than it does in HOA. In a similar task, a compensation system that included the amygdala was retrieved, as shown when greater amygdala activation was reported in patients with mild cognitive impairments compared with HOA (pilot study reported in: Broster et al., 2012). This result corroborates findings of Wright et al. (2007), in which amygdala activity was shown to be significantly greater in AD patients for both neutral and emotional faces compared with HOA. The hypothesis that the preservation of emotional impact might be explained by a compensatory mechanism requiring greater recruitment of the amygdala and/or solicitation of an additional anatomical network thus seems valuable (Grady et al., 2001; Wright et al., 2007; Rosenbaum et al., 2010; Broster et al., 2012). Further studies investigating the functional interactions between prefrontal and amygdala areas in the context of emotional memory in AD are needed before conclusions may be drawn.

**Figure 1:**
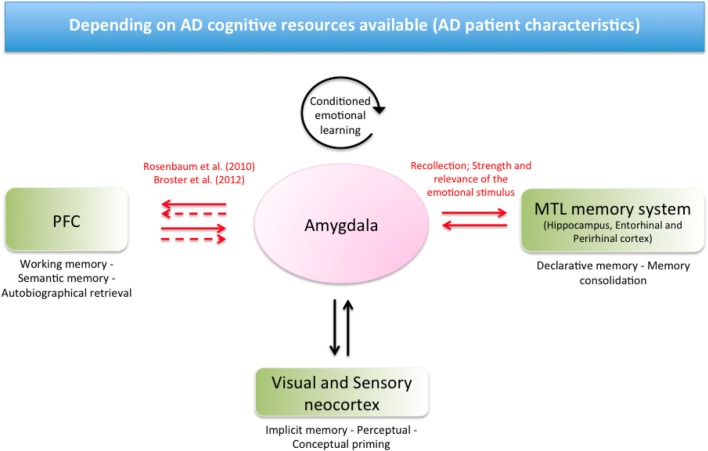
**The model based on potential mechanisms by which the amygdala mediates the influence of emotional effect on memory in AD, inspired by Labar and Cabeza (2006)**. The amygdala presents neural projections to several brain areas. In the context of AD, we propose a pattern of preserved or reinforced interactions between the amygdala and MTL structures in the context of successful emotional retrieval in declarative memory (potentially depending on the task and the emotional stimulus). A pattern of preserved or reinforced interactions between the amygdala and prefrontal cortex could act in the context of flashbulb memory, one form of autobiographical memory. Compensatory mechanisms have also been proposed in the context of working memory (Rosenbaum et al., 2010; Broster et al., 2012). On the other hand, non-declarative memory could be impaired in the context of fear conditioning, as this system takes place intrinsically in the amygdala. Emotional priming could be preserved, as visual and sensory cortices remain relatively preserved in AD, leading to potentially intact neural projections to the amygdala. The EEE requires that cognitive resources are available to help performance for a given task and for a given participant. Red arrows indicate a pattern of reinforced neural projections. Solid arrows indicate direct connections; dashed arrows indicate indirect connections. PFC, Prefrontal cortex; MTL, Medial temporal lobe; AD, Alzheimer’s disease.

In another model, Labar and Cabeza (2006) emphasized the role of the amygdala and its interaction with other brain areas, including MTL structures and the prefrontal cortex, in the context of declarative and non-declarative memory. In this theoretical framework, the amygdala mediates the influence of emotional arousal on declarative memory via direct connections with the MTL structures by favoring memory consolidation. Indirect and direct connections between the prefrontal cortex and the amygdala mediate other forms of declarative memory, including semantic, autobiographical, and working memory. In addition to the conditioned emotional learning that takes place intrinsically in the amygdala, direct neural projections with sensory cortices target other non-declarative forms of memory, including perceptual and conceptual priming [see Figure 1, inspired by Labar and Cabeza (2006)].

As stated above in the context of AD and on the basis of this model, we hypothesize that a patient’s successful recollection of emotional cues (vs. non-emotional cues) could relate to preserved or reinforced functional interactions between the amygdala and MTL structures in the context of declarative memory. These amygdala and MTL interactions for EEE elicitation could also depend on the arousal strength and relevance of the emotional stimulus (Ikeda et al., 1998; Satler et al., 2007; Sundstrom, 2011; Kalenzaga et al., 2012). In the case of enhanced flashbulb memory, one form of autobiographical memory, additional reinforced projections between the amygdala and prefrontal cortex could be recruited to enhance memory, providing that the emotional stimulus is strong enough (Mori et al., 1997; Ikeda et al., 1998; Budson et al., 2004). The model of Labar and Cabeza (2006) also fits with suggestions of Broster et al. (2012) and Rosenbaum et al. (2010) on the potential compensatory mechanism involving the prefrontal cortex and the amygdala in the context of working memory (studies involving match-to-sample tasks). Considering non-declarative memory, we hypothesize that the later impairments of sensory cortices could result in potentially intact projections between the amygdala and this area to maintain an emotional influence on priming scores. On the other hand, additional brain networks cannot compensate for fear learning impairments in AD, which mainly involve the amygdala.

Neuroimaging of emotional memory enhancement in aging and AD populations remains in its infancy (Broster et al., 2012). The model that we propose in the context of AD is based on current knowledge of brain networks that sustain emotional memory processes in healthy subjects (Labar and Cabeza, 2006). The AD population highly differs from normal subjects in the sense that patients have severe cognitive declines. Thus, our assumptions of reinforced neural projections between brain areas in the elicitation of emotional enhancement have to be considered in the context of cognitive resources that are available in the AD population (Borg et al., 2011; Broster et al., 2012). In this way, severe deficits in the overall memory system or executive functions could compromise an emotional effect on memory for a given task.

This AD model of neuropathological changes provides new input into the current staging of knowledge concerning emotional memory processes in humans. There may not be a linear explanation for the relation between amygdala volumes, its functional activity, and emotional memory disturbances. Further neuroimaging findings would be very helpful in describing the anatomical and functional signatures of emotional memory processes and how altered brain systems may compensate for emotional memory impairments in the context of AD.

## Conflict of Interest Statement

The authors declare that the research was conducted in the absence of any commercial or financial relationships that could be construed as a potential conflict of interest.
